# Scratch Properties of Clear Coat for Automotive Coating Comprising Molecular Necklace Crosslinkers with Silane Functional Groups for Various Environmental Factors

**DOI:** 10.3390/polym13223933

**Published:** 2021-11-14

**Authors:** Sung Wook Moon, Jiae Seo, Ji-Hun Seo, Byoung-Ho Choi

**Affiliations:** 1 School of Mechanical Engineering, College of Engineering, Korea University, 145 Anam-ro, Seongbuk-gu, Seoul 02841, Korea; mlshine99@korea.ac.kr; 2 Department of Materials Science and Engineering, Korea University, 145 Anam-ro, Seongbuk-gu, Seoul 02841, Korea; sja920430@korea.ac.kr (J.S.); seojh79@korea.ac.kr (J.-H.S.)

**Keywords:** scratch, automotive coat, polyrotaxane, silane functional group, high temperature, ultraviolet

## Abstract

Automotive coatings, which comprise multiple layers, i.e., primer, base coating, and clear coat layers, are exposed to various environmental conditions that pose various types of damages to them. In particular, the outer layer of the automotive coating, i.e., the clear coat, is affected significantly by such damages. Therefore, a reliable and durable clear coat must be developed to improve the appearance of automobiles. In this study, a new clear coat based on an acrylic-based clear coat modified using polyrotaxane crosslinkers, which are necklace-shaped supramolecules composed of ring-shaped host molecules, is developed and characterized. The effects of polyrotaxane and silane on the scratch properties and mechanisms of the clear coating are analyzed. It is observed that the critical loads of the clear coat from scratch tests can be improved by adding optimal molecular necklace crosslinkers comprising silane functional groups. The improvement in the scratch properties of the modified acrylic-based clear coat may be attributed to the crosslinking characteristics and dynamic molecular movements of the polyrotaxane. In addition, the effects of environmental factors on the scratch characteristics of the modified acrylic-based clear coat are investigated by addressing the scratch durability of the clear coat.

## 1. Introduction

Automotive coatings can be regarded as key in preventing various damages, e.g., scuffing, scratching, and chipping, in order to maintain the initial esthetics and afford stable functionality. The coating is a multilayer coating on electrodeposited zinc-plated steel that has undergone phosphoric acid treatment, whose function is to increase adhesion and improve corrosion protection. It comprises a primer surface (35–40 mm), a base coating layer (10–15 mm, which is the colored layer), and a clear coat (35–40 mm) [1,2,3]. This multilayer coating film has been developed to enhance resistance to physical–mechanical damage by reinforcing the properties of the coating material. According to research trends pertaining to automotive coats hitherto, characteristics of existing paint, research regarding adhesion between substrate and coating, and improvement in paint processes are garnering interest. Recently, organic–inorganic hybrid clear coats have been employed to improve durability. Through the organic–inorganic hybridization of the clear coat, the resistance of the clear coat to physical damage can be improved by modifying a clear coat that possesses the advantages of organic materials (flexibility and elasticity, etc.) and inorganic materials (strong hardness, etc.) simultaneously [4,5,6]. However, in many cases, inorganic materials degrade the clarity of the clear coat, and such approaches are difficult to commercialize in the automotive industry.

It is important to first identify the scratch damage mechanism to improve the scratch resistance of a clear coat. The polymer scratch damage mechanism varies in a sporadic manner, owing to the critical loads under various environmental and operating conditions. Therefore, the damage mechanism significantly affects the degree of damage to the clear coat; as such, the critical load should be delayed when possible [7]. In automotive coatings, most scratches on the surface are caused by damage to the clear coat. This can result in consumer complaints, who perceive the scratches as actual damage. Therefore, improving the scratch resistance of a clear coat is critical to ensure customer satisfaction [8].

In this study, to improve the scratch resistance of the clear coat, a clear coat structure was modified using a crosslinking agent, and a silane functional group was introduced into polyrotaxane. Polyrotaxane is a necklace-shaped supramolecule composed of ring-shaped host molecules, where α-cyclodextrin (α-CD) is threaded onto a linear guest molecule. Poly(ethylene glycol) (PEG) and 10–80 α-CD molecules can be threaded into a single PEG chain with a molecular weight of 10,000 [9,10,11,12]. Because six primary hydroxyl groups and 12 secondary hydroxyl groups exist in a single α-CD molecule, 180–1440 reactive groups can be potentially introduced into PEG with a molecular weight of 10,000, which is an ideal molecular structure that maximizes the crosslinking density of the polymer network [13,14,15]. Furthermore, crosslinked α-CD molecules are expected to exhibit dynamic motion along the PEG axis under physical stretching because polyrotaxane is a mechanochemically synthesized molecule that is not bonded covalently [16,17]. Therefore, the polyrotaxane-based hard coating material is expected to exhibit high scratch resistance owing to the formation of a crosslink network and the flexible properties derived from the movable crosslink point. Therefore, in this study, acrylic-based clear coats modified using a molecular necklace crosslinker were developed; this can simultaneously increase scratch resistance and flexibility owing to the crosslinking characteristics and dynamic molecular motion. In addition, scratch experiments were conducted under high-temperatures and ultraviolet (UV) irradiation conditions, and the scratch characteristics of the developed clear coat were evaluated comparatively.

## 2. Materials and Test Methods

In this study, three types of specimens with acrylic-based clear coats were fabricated. The first specimen type, which is named a “clear-coat specimen”, was a commercial automotive coating specimen with 8% polyester added to an acrylic-based clear coat paint TT6832 provided by KCC (Seoul, Korea). This specimen was a reference specimen prepared by coating the primer, base coat, and clear coat on the base metal as a conventional automobile multilayer coating. The second specimen type, which is named a “B-clear specimen”, was prepared using a bar coater by coating the primer on the base metal without a base coat and then applying the commercial clear coat used for the first specimen type. In the first and second specimen types, the scratch properties of the clear coat were compared, and it was verified that a clear coat can be prepared on the first specimen type using a bar coater, similar to the second specimen type. The third specimen type, which is named a “PRX-Si specimen”, was prepared using a bar coater by applying a newly developed clear coat modified with 5 wt% polyrotaxane comprising silane functional groups to the conventional clear coat paint TT6832 with 8% polyester. For the PRX-Si specimens, 1, 2, 3, and 5 silane functional groups per α-CD molecule were introduced, and they were named PRX-Si1, 2, 3, and 5, respectively.

Because automobiles are exposed to various weathering environments, the durability of the clear coats must be ensured. In particular, the degradation of clear coats under high temperatures and UV irradiation environments must be understood. Therefore, in this study, a comparative analysis was performed after the clear coats were exposed to high temperatures and UV irradiation. The average annual maximum temperature in Seoul is 36.6 °C. The temperature of automotive coating differs based on the base coat color; for example, for a midnight-black base coat color, the measured temperature was 83.3 °C. In this experiment, exposure at 100 °C for 48 h in an oven was implemented, which is a harsh high-temperature condition [18]. UV irradiation is an important factor that significantly affects the automotive coat, such as its temperature. A xenon-arc ramp (model number UXL-3000HK-O) from USHIO Korea (Seoul, Korea) with light properties similar to sunlight was used to determine the effect of UV light. Based on SAE J 1960, the specimen was fixed on a circular plate rotating at a constant angle of 0.2 rad/s and exposed to 61 MJ/m^2^ of UV light for 400 h [19,20,21,22]. This was calculated based on 3.5 years of exposure using the SAE standard; however, the SAE standard repeats exposure and non-exposure to UV light for a certain duration. However, in this study, UV exposure was continued for 400 h, which was calculated to be approximately 6 years of exposure.

The equipment used for the experiment was the scratch tester KK-01 from Kato Tech (Kyoto, Japan), which can record the normal load, horizontal load, and scratch distance, and the experiment was conducted in accordance with ASTM D 7027 [23]. The experimental method was performed in two modes, i.e., Modes A and B. Mode A identifies the characteristics of automotive coatings based on changes in the tip diameter and the scratch speed under a constant normal load. In this study, Mode A was conducted while changing the constant normal load at intervals of 10 N within a range from 20 to 100 N. In Mode B, the experiment was conducted while increasing the normal load to establish the damage mechanism of the coating film based on the speed and diameter of the tip, and then the peeling properties were evaluated. At least three experiments were performed to maintain the reproducibility for each experimental condition. Detailed experimental methods are summarized in Table 1. The damaged samples were observed via optical microscopy to determine the relative macroscopic damage mechanisms and damage transition points. This reveals the differences between the samples, thereby allowing a transition point to be defined. Each of these transition points is the starting point for the visualization of the scratch, and the normal load at each transition point indicates the critical load. In fact, the higher the critical load, the higher the scratch resistance.

Glossiness, hardness, and degradation were measured to confirm the physical properties of the specimens based on each condition. Glossiness was measured five times using a glossiness tester GM-268 from LANDTEK (Guangzhou, China), in accordance with the ASTM D 523 standard [24]. Hardness was measured five times using an O.M.A.G ART13 from AFFRI Inc. (Wood Dale, IL, USA). The degradation degree was measured using the Diamond ATR version of the Cary 630 FT-IR equipment from Agilent Technologies (Santa Clara, CA, USA).

## 3. Test Results and Discussions

### 3.1. Scratch Characteristics of Commercial Automotive Coat

**Figure 1:**
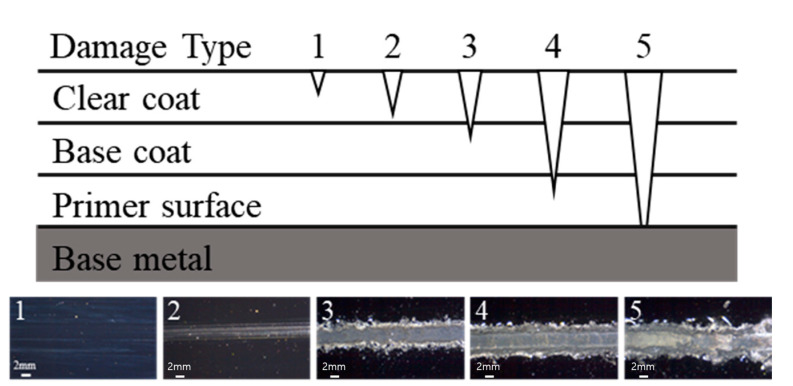
Scratch damage types of automotive coating identified using 1 mm tip at scratch speed of 100 mm/s.

As shown in Figure 1, the typical scratch characteristics of automotive coats were classified based on damage mechanisms using clear-coat specimens. The effects of the constant normal load, tip size, and scratch speed based on Mode A on the scratch characteristics of the coating film were investigated. The photograph at the bottom of Figure 1 shows the variation in the scratch damage morphologies obtained by changing the normal load using the 1 mm tip at a scratch speed of 100 mm/s. Damage type 1 refers to scratches in the mar mode without significant scratch damages for loads below 20 N. Damage type 2 shows visible scratch damage behaviors with whitening in the clear coat caused by 20–30 N loads. Damage type 3 is caused by the delamination between the clear coat and base coat at 30 N. Damage type 4 refers to scratches caused by base coat delamination from the primer surface at 40 N. Damage type 5 refers to the complete delamination of the multilayer coating at 80 N, in which the base metal is eventually revealed.

**Figure 2:**
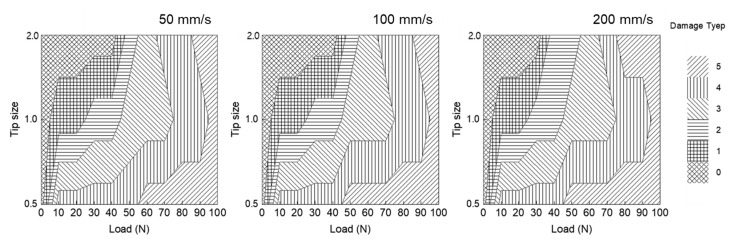
Damage mechanism map based on Mode A scratch tests for various scratch speeds and tip size.

Figure 2 shows the damage type based on the tip size and speed. As shown, the graph trend did not change even when the scratch speed increased; this implies that the scratch damage type did not significantly affect the speed. By contrast, when the tip size was increased, the occurrence of scratches was delayed. Hence, it can be inferred that the effect of the tip on the scratch was greater than that under the same load.

**Figure 3:**
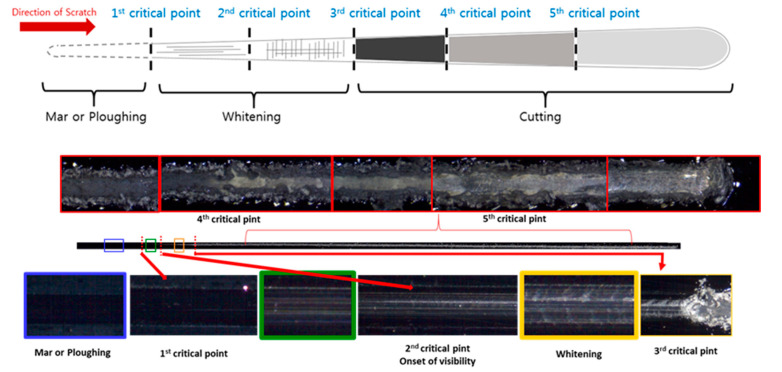
Scratch mechanism evolution of automotive multilayer coating [8].

The change in the damage mechanism of the automotive coat based on the load change was confirmed through Mode B. Similar to the Mode A scratch test, five damage transition points were observed, and these transition points show that the scratch damage mechanism changed discontinuously; therefore, they are known as critical points. The details of the scratch damage evolving with the critical points are schematically summarized and shown in Figure 3. Initially, damage type 1 with minimized scratch damage, i.e., mar damage in the clear coat, occurred as the load increased until the first critical point. After traversing the first critical point, the scratch damage mechanism is changed to damage type 2, i.e., whitening in the clear coat, until the second critical point. Therefore, it is assumed that the first critical load is closely associated with the onset of the visibility point, where scratches are visible to the naked eye. After the Mode 2 damage occurred, transition points were observed whenever multilayered interlayer delamination occurred. In this case, the third critical point is where the clear coat and base coat are delaminated, and the fourth critical point is where the base coat and primer are delaminated. Finally, the fifth critical point refers to the point where the primer and base metal are separated.

**Figure 4:**
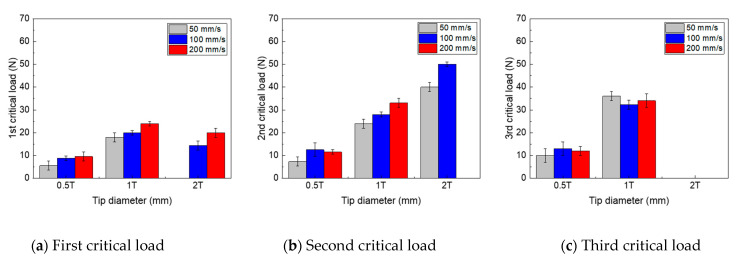
Critical loads of multilayer coat at various scratch speeds and tip sizes.

Among the five critical loads, the transition point associated with the scratch damage of the clear coat can be evaluated based on the first three critical loads. The degree of scratch damage of the clear coat based on the scratch speed and tip size was compared, as shown in Figure 4. It was observed that the first and second critical loads increased with the scratch speed, owing to the stiffening effect of the clear coat at higher scratch speeds. However, the third critical load, which is associated with the delamination between the clear and base coats, was not significantly affected by the scratch speed. In addition, for the 2 mm tip, the third and second critical loads at a scratch speed of 200 mm/s were not observed within the normal scratch load range of this study. An increase in the scratch tip size corresponds to a decrease in the local stress required for scratch initiation. Therefore, it was observed that all the critical loads increased with the scratch tip size. However, when the experiment was conducted using a 2 mm scratch tip at a scratch speed of 50 mm, the scratch damage was minimal; therefore, mar or plowing damage associated with the first critical load was not apparent.

**Figure 5:**
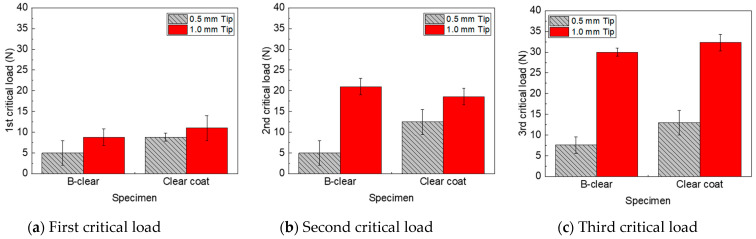
Comparison of scratch critical loads of commercial clear coat and B-clear specimens for various tip sizes.

In this study, multilayer coated specimens were fabricated with a primer on a base metal without a base coat to evaluate the scratch properties of a newly developed clear coat modified using polyrotaxane comprising a silane functional group. To examine whether the scratch characteristics of the clear coat of specimens with and without the base coat changed, the scratch characteristics between a commercially available clear-coat specimen and the B-clear specimen without a base coat fabricated via the process described above were compared. The clear-coat and B-clear specimens exhibited similar glossiness and Shore hardness results. In the experiment, the scratch speed was set to 100 mm/s, and 0.5 mm and 1 mm tips were used based on Mode B. The 2 mm tip was excluded because it did not sufficiently reflect scratch damages, based on the test results of clear-coat specimens. Figure 5 shows the scratch test results for the clear-coat and B-clear specimens. The fabrication conditions of the commercial clear coat were more optimized than those of the B-clear specimen; therefore, the scratch properties of the B-clear specimen were evaluated as inferior compared with those of the clear-coat specimen. However, compared with the scratch-sensitive 0.5 mm tip, when the 1 mm tip was used, the variation in critical loads between the two specimens was slight. Therefore, it was inferred that using the 1 mm tip is appropriate for investigating the scratch characteristics of the new clear coat. Therefore, the scratch test conditions for the newly developed clear coat were determined based on Mode B (1 mm tip, 100 mm/s of scratch speed, and progressively increasing normal load from 2 to 100 N). Additionally, the first, second, and third critical points were measured and analyzed.

### 3.2. Scratch Characteristics of PRX-Si Specimen

#### 3.2.1. Effect of Modifying Polyrotaxane with Silane Functional Groups

**Figure 6:**
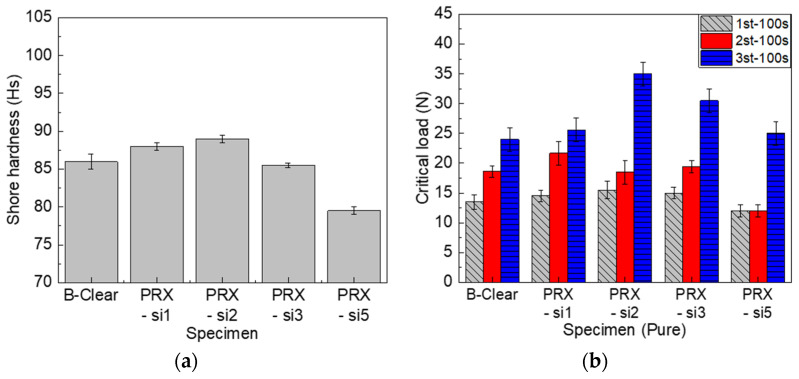
Effect of polyrotaxane comprising silane functional groups on Shore hardness (**a**) and scratch critical loads (**b**) of PRX-Si and B-clear specimens.

The PRX-Si specimens, which contain polyrotaxane with silane functional groups, did not differ from the conventional B-clear specimen in terms of glossiness. It was discovered that the silane functional groups in polyrotaxane did not significantly affect the optical properties of the PRX-Si specimens, and the new clear coat material modified by polyrotaxane comprising a silane functional group can be commercially applied to automotive coatings. This result is different from that achieved by introducing inorganic fillers [25], which have been widely applied to improve scratch resistance in the past, although practicality was not ensured owing to a decrease in glossiness caused by hazing. In addition, as shown in Figure 6a, the clear coat modified by the silane functional groups in polyrotaxane increased the Shore hardness, which then decreased. This is speculated to be due to the structural singularity of the molecular necklace-like PRX; an excessive number of silane functional groups will cause embrittlement, which implies that the unique mechanical properties will deteriorate gradually. In addition, when the number of silane functional groups is excessive, as in the PRX-Si10 test piece, the hardness increases because of the increase in the crosslinking effect, but the clear coat becomes embrittled [26], rendering it difficult to satisfy the physical and mechanical properties required of the clear coat. Therefore, these results indicate that the use of polyrotaxane derivatives with an optimized composition of the silane functional groups of polyrotaxane is important to achieve a balance between mechanical flexibility and enhanced mechanical strength [17]. Figure 6b shows the scratch evaluation results for the PRX-Si and B-clear specimens. Similarly to the test results of the Shore hardness, it was confirmed that the first critical load increased up until a certain number of silane functional groups, and then decreased, owing to the embrittlement of the clear coat. In addition, the second critical load was measured as being almost identical to the B-clear specimen up until a certain number of silane functional groups. However, it was observed that the scratch characteristics of the PRX-Si5 and PRX-Si10 specimens deteriorated. Because the first and second critical loads were associated with the local damage and scratch visibility of the clear coat along the scratch direction, it was confirmed that the onset of visible scratch damage was delayed by the introduction of polyrotaxane comprising silane functional groups. This is because more silanes had formed in the polyrotaxane crosslinking agent, causing more glass crystals to be formed and the scratch resistance to external force to increase. By contrast, when many functional groups were formed, the polyrotaxane crosslinking agents aggregated, yielding the effect of an inorganic additive. In addition, the same trend was observed in the case involving the third critical load. This may indirectly explain the interfacial characteristics and indicate that polyrotaxane comprising silane functional groups can improve the interfacial characteristics of multilayer coatings.

#### 3.2.2. Effect of Thermal Degradation

**Figure 7:**
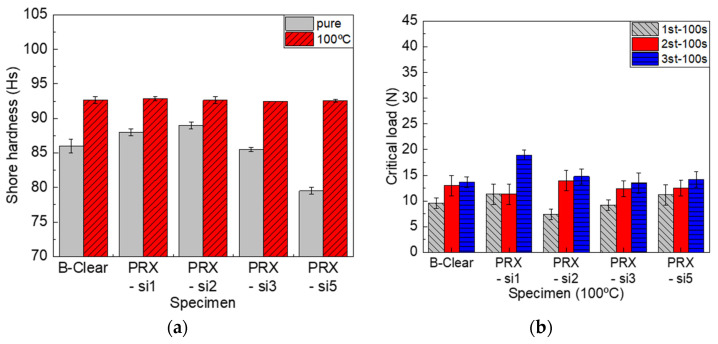
Effect of thermal degradation at 100 °C for 48 h on Shore hardness (**a**) and scratch critical loads (**b**) of B-clear and PRX-Si specimens.

To understand the thermal stability of the newly developed clear coat, specimens exposed to 100 °C for 48 h were analyzed. After UV irradiation, the glossiness of the B-clear specimen decreased slightly, whereas the glossiness of the PRX-Si specimen decreased by approximately 10%. In addition, as shown in Figure 7a, an increase in the Shore hardness was observed in all specimens owing to thermal deterioration. Because the conditions of 100 °C and 48 h do not reflect those at which the polyrotaxane polymer is thermally decomposed, it is assumed that the added polyrotaxane will not be thermally decomposed. However, it was clear that the structure of the clear coat material might change, owing to reasons such as the crosslinking effect and oxidation due to thermal degradation. As shown in Figure 7b, the scratch characteristics changed significantly because of thermal degradation. Although both the first and second critical loads decreased as thermal degradation progressed, it was observed that the decrease was greater in the PRX-Si specimens than that in the B-clear specimens. Polymers that were modified with a small amount of silane functional groups, such as the PRX-Si1 specimen, did not degrade significantly because the flexibility of the clear coat was maintained, owing to the low crosslinking degree. However, in terms of the PRX-Si2 and PRX-Si3 specimens, their flexibility deteriorated because the crosslinking density increased. Therefore, it was assumed that embrittlement due to thermal degradation significantly deteriorated the scratch properties. In terms of the PRX-Si5 specimen, whose crosslinking agent was substituted with a significant amount of silane, it was speculated that thermal degradation was limited by the self-crosslinking effect, owing to the significant number of silane functional groups. In particular, under thermal degradation, the third critical load reduced significantly, unlike the first and second critical loads. This implies that the delamination between the clear coat and other coating layers can be accelerated, owing to long-term deterioration as well as a deterioration in the scratch properties of the clear coat.

**Figure 8:**
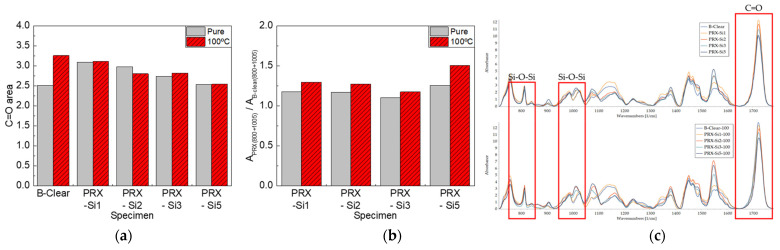
FT-IR analysis results of B-clear and PRX-Si specimens under thermal degradation conditions. (**a**) Variation of C=O peak area, (**b**) Variation of normalized Si-O-Si peak areas, and (**c**) Comparison of FT-IR spectra.

In the commercial automotive clear coats, variation in the peak intensity of some specific wavenumbers was detected, based on FT-IR analysis. In particular, the band at 1720 cm^−1^ corresponded to a C=O formation peak due to degradation, whereas that at 1680 cm^−1^ corresponded to a progressive degradation peak [27,28,29,30,31,32]. As shown in Figure 8a, the change in the C=O peak area was more significant in the B-clear specimen as compared with that in the PRX-Si specimen. As described above, it was speculated that this phenomenon occurred because of the crosslinking level of the PRX-Si specimens. Nevertheless, as shown in Figure 7, the hardness of the PRX-Si specimen increased under thermal degradation conditions, and the physical properties of the scratches degraded. To further examine this phenomenon, the variation in crosslinking characteristics was considered. In the B-clear specimens, thermal degradation, associated with the increase in the number of C=O peaks, might have increased the hardness. Meanwhile, in terms of the PRX-Si specimens, it was observed that the variation occurred in the Si–O–Si bonds, based on the area of the 800 and 1005 cm^−1^ bands of the PRX-Si specimen normalized by that of the undegraded B-clear specimen, as shown in Figure 8b. It was assumed that this change in crosslinking properties was associated with an increased number of crosslinking points, decreased flexibility, and increased hardness and strength. In addition, as the number of Si–O–Si bonds increased due to thermal degradation, air bubbles were generated in the PRX-Si specimen, indicating reduced glossiness.

**Figure 9:**
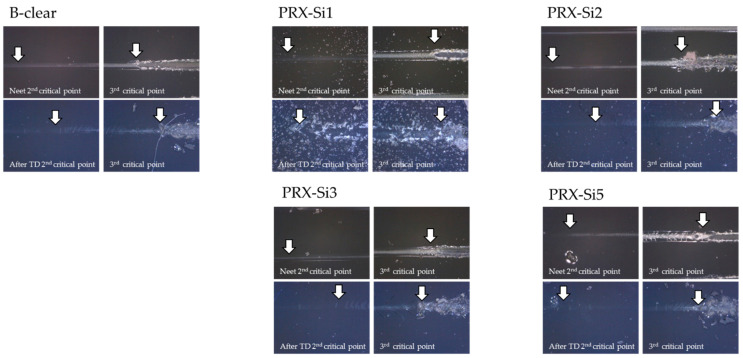
Variation and observation of scratch damage mechanisms around 2^nd^ and 3^rd^ critical loads under thermal degradation conditions.

As shown in Figure 9, the scratch damage mechanisms changed depending on the thermal degradation. Figure 9 shows the second and third critical points of the B-clear and PRX-Si specimens before and after thermal degradation. Based on the thermal degradation in all specimens, the undegraded clear coat indicated less brittleness due to the scratch damage as compared with the other specimens. Compared with the B-clear specimen, the PRX-Si specimen showed a similar scratch mechanism change as compared with the whitening mode after the second critical load, but the scratch damage was less visible. This may be because, in the PRX-Si specimen, the degradation due to crosslinking was dominant during thermal degradation, which resulted in less embrittlement during scratching as compared with the B-clear specimen. In addition, in the case involving the 3^rd^ critical load, because delamination between coating layers accelerated owing to thermal degradation, it was observed that local delamination occurred before degradation. However, scratch damage with no scratch shoulder occurred and clear coat fragments appeared in more areas of the thermally degraded specimens compared with neat specimens.

#### 3.2.3. Effect of UV Degradation

**Figure 10:**
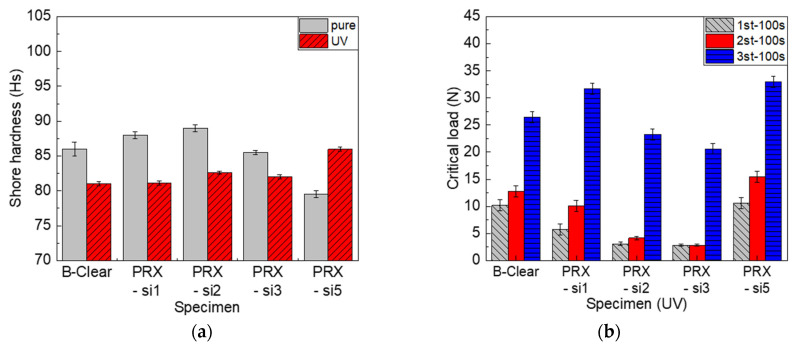
Effect of UV photodegradation with 61 MJ/m^2^ UV irradiation for 400 h on Shore hardness (**a**) and scratch critical loads (**b**) of B-clear and PRX-Si specimens.

An important factor affecting the durability of automotive clear coats along with thermal degradation is UV irradiation [33,34,35,36]. In the case of automotive multilayer coating, UV degradation is important mainly for clear coats rather than other coating layers. Under the aforementioned UV irradiation conditions, the glossiness decreased by approximately 10%, similar to the result of thermal degradation in all specimens. However, in terms of hardness and scratch properties, the thermal degradation and UV degradation showed significantly different tendencies. Figure 10 shows the changes in the Shore hardness and scratch critical loads of the B-clear and PRX-Si specimens in terms of UV irradiation. As shown in Figure 10a, unlike the trend for thermal degradation, the Shore hardness decreased in all specimens except for specimen PRX-Si5, which exhibited high crosslinking characteristics. In addition, as shown in Figure 10b, the scratch critical loads reduced significantly, except for the PRX-Si specimen. This is speculated to be due to a change in the crosslinking properties of the clear coat material owing to photodegradation by UV irradiation. In addition, the third critical load indicated an unusually high value, unlike the significant decrease observed in the other critical loads. This was speculated to be caused by the confinement of the UV degradation to the surface owing to the short wavelength of UV light. Unlike the first and second critical loads, which are closely associated with surface degradation, the third critical load associated with delamination between the coating layers was affected less by UV irradiation.

**Figure 11:**
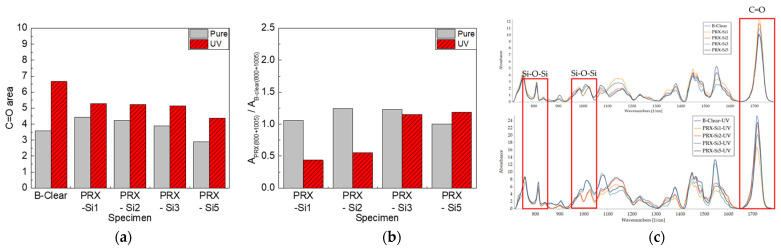
FT-IR analysis results of B-clear and PRX-Si specimens under UV photodegradation conditions. (**a**) Variation of C=O peak area, (**b**) Variation of normalized Si-O-Si peak areas, and (**c**) Comparison of FT-IR spectra.

Similar to the effect of thermal degradation, changes in the chain structure due to UV irradiation were analyzed using FT-IR. As shown in Figure 11a, the C=O area decreased in both the B-clear and PRX-Si specimens, unlike the thermal degradation caused by the degradation of the clear coat due to UV irradiation. It can be inferred that photodegradation due to UV irradiation resulted in chain scission in the PRX-Si specimen. Meanwhile, as shown in Figure 11b, the areas of the 800 and 1005 cm^−1^ bands normalized to the area of the undegraded B-clear specimen and were silane functional for the PRX-Si specimen. However, for the PRX-Si1 and PRX-Si2 specimens with few silane functional groups, the normalized areas of the 800 and 1005 cm^−1^ bands decreased. However, for the PRX-Si3 and PRX-Si5 specimens comprising many silane functional groups, the normalized areas of the 800 and 1005 cm^−1^ bands decreased only slightly. When only a few silane functional groups were present because of UV irradiation, the clear coat became brittle primarily because of chain scission. By contrast, for the PRX-Si5 specimen, an effect similar to that of inorganic silica particles occurred via a self-condensation reaction instead of via crosslinking with a clear coat. Therefore, the degradation of the clear coat was slight, and chain scission was less frequent; hence, the reduction in the scratch critical loads was less.

**Figure 12:**
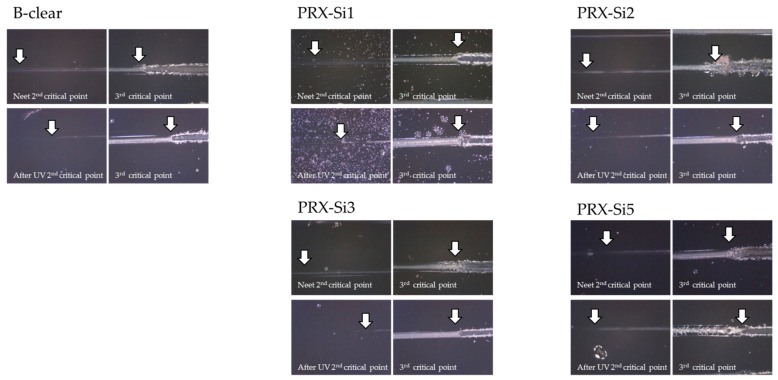
Variation in scratch damage mechanisms for second and third critical loads under UV photodegradation conditions.

The change in the scratch mechanism due to deterioration in UV irradiation was observed under an optical microscope, and the results are shown in Figure 12. In the PRX-Si1 and PRX-Si2 test pieces, similar to the B-clear test piece, the surface hardness decreased owing to deterioration in UV irradiation, and the scratch damage mechanism changed similarly to that of soft materials. However, this phenomenon diminished gradually as the number of silane functional groups increased. In the PRX-Si5 specimen, almost no chain scission occurred and a certain level of crosslinking was maintained; therefore, the scratch damage mechanism changed only slightly. In addition, it was observed that the clear coat had peeled off with sufficient plastic deformation without exhibiting significant brittle damage, unlike the thermal deterioration before and after the third critical load, which is associated with delamination between coating layers.

## 4. Conclusions

In this study, a new acrylic-based clear coat, modified using polyrotaxane, which is a necklace-shaped supramolecule composed of ring-shaped host molecules with α-CD threaded onto a linear guest molecule, and a durable and reliable automotive coating composed of silane functional groups, were developed and characterized. The conclusions of this study are as follows:

(1) A total of five scratch mechanisms were identified by evaluating the constant load scratch characteristics of commercial automotive multilayer coatings, and a scratch damage mechanism map was constructed by changing the scratch tip size and scratch speed. Based on a progressive scratch test, the commercial vehicle multilayer coating damage mechanism was evaluated based on five critical loads, where the first, second, and third critical loads were changed to evaluate the scratch properties of the clear coat by changing the scratch tip size and scratch rate. In addition, to evaluate the scratch properties of the new clear coat material, a B-clear specimen was prepared, and the scratch test conditions were determined based on the effectiveness of the B-clear specimen.

(2) A PRX-Si specimen, which was applied with a new clear coat material achieved by modifying a commercially available clear coat material with 5 wt% polyrotaxane comprising silane functional groups, was prepared in the same manner as the B-clear specimen. The new PRX-Si specimen exhibited glossiness similar to that of the existing commercial clear coat. It was observed that the PRX-Si2 specimen yielded the best Shore hardness and scratch properties by changing the silane functional groups.

(3) The thermal degradation characteristics of the novel PRX-Si specimen were examined, and it was confirmed that the Shore hardness increased with thermal degradation, but the scratch critical loads decreased. Through FT-IR analysis, it was observed that fewer chain scissions occurred in the PRX-Si specimen as compared with the B-clear specimen; however, crosslinking was promoted in the silane functional groups of the PRX-Si specimen due to thermal degradation, which limited the flexibility of the polyrotaxane. It was assumed that the brittle scratch characteristics were expressed.

(4) The degradation behavior of the novel PRX-Si specimen due to UV irradiation was evaluated, and, unlike the thermal degradation trend, all but the PRX-Si5 specimen indicated a decrease in the Shore hardness and a significant decrease in the first and second critical loads. This was concluded as the main cause of chain scission due to UV photodegradation. In the PRX-Si5 specimen with many crosslinking points, the crosslinking degree was maintained, and the rapid change in the scratch properties was minimized. In addition, it was observed that, unlike thermal degradation, degradation due to UV irradiation was limited to the surface because of the short wavenumber of UV light. Hence, the third critical load was barely affected.

## Figures and Tables

**Table 1:** Scratch test mode and test conditions.

Mode	Tip diameter (mm)	Scratch speed (mm/s)	Scratch length (mm)	Temperature (°C)	Number of Scratches
Mode A	0.5, 1, 2	50, 100, 200	100	Room	3
Mode B	0.5, 1, 2	50, 100, 200	100	Room	3

## Data Availability

The data presented in this study are available on request from the corresponding author.
